# Overexpression of CREMαlpha accelerates onset and severity of auto-immune mediated disease in a murine model of lupus

**DOI:** 10.1186/1546-0096-9-S1-O3

**Published:** 2011-09-14

**Authors:** Kim Ohl, Ralph Lippe, Eva Verjans, Nora Honke, Karin Maschke-Neuss, Norbert Wagner, Johannes Roth, Klaus Tenbrock

**Affiliations:** 1 Klinik für Kinder und Jugendmedizin, Klinikum der RWTH Aachen, Germany; 2 Institut für Immunologie, Universität Münster, Germany

## 

The transcription factor cAMP response element modulator (CREM) is a widely expressed transcriptional repressor which is important for the termination of the T cell immune response. CREMα is overexpressed in SLE (Systemic lupus erythematosus) T cells and is supposed to be a key player in orchestrating the transcriptional program of SLE T cells by targeting T cell-relevant genes. To explore the relevance of CREMα in vivo we used a well-established murine lupus model, which is characterized by the introduction of a mutation in the CD95 (Fas) locus. We generated a transgenic mouse with a selective overexpression of CREMα in T cells and introduced a Fas -/- phenotype into the CREMα transgenic mice. CREMα transgenic Fas -/- mice developed a severe lymphadenopathy and splenomegaly as early as 8 weeks of age, while the wildytpe Fas -/- mice did not at this early age. Lymphadenopathy and splenomegaly is paralleled by a massive expansion of pathogenic CD3+CD4-CD8- double negative T cells. Furthermore T cells of CREMα transgenic Fas -/- mice show an enhanced production of IL-21 and IL-17, which are hallmark cytokines of highly inflammatory Th17 cells. Vice versa percentages of regulatory T cells are reduced. The enhanced occurrence of aberrant and inflammatory T cells further leads to increased B cell activation, increased anti-DNA antibody titers and finally shortened life expectation of these mice.

Our experiments are the proof of principle for a critical amplifying role of CREMα in autoimmune prone conditions like SLE.

